# A meta-analysis to assess long-term spatiotemporal changes of benthic coral and macroalgae cover in the Mexican Caribbean

**DOI:** 10.1038/s41598-020-65801-8

**Published:** 2020-06-01

**Authors:** Ameris I. Contreras-Silva, Arjen Tilstra, Valentina Migani, Andra Thiel, Esmeralda Pérez-Cervantes, Nuria Estrada-Saldívar, Xochitl Elias-Ilosvay, Claudius Mott, Lorenzo Alvarez-Filip, Christian Wild

**Affiliations:** 1 Marine Ecology Department, Faculty of Biology and Chemistry, University of Bremen, Leobener Straße UFT, 28359 Bremen, Germany; 2 Population and Evolutionary Ecology Group, Institute of Ecology, Faculty of Biology and Chemistry, University of Bremen, Leobener Straße 5, 28359 Bremen, Germany; 3 Biodiversity and Reef Conservation Laboratory, Unidad Académica de Sistemas Arrecifales, Instituto de Ciencias del Mar y Limnología, Universidad Nacional Autónoma de México, Puerto Morelos, Quintana Roo Mexico; 4 Remote Sensing Solutions GmbH, Dingolfinger Str. 9, 81673 München, Germany

**Keywords:** Tropical ecology, Marine biology

## Abstract

Coral reefs in the wider Caribbean declined in hard coral cover by ~80% since the 1970s, but spatiotemporal analyses for sub-regions are lacking. Here, we explored benthic change patterns in the Mexican Caribbean reefs through meta-analysis between 1978 and 2016 including 125 coral reef sites. Findings revealed that hard coral cover decreased from ~26% in the 1970s to 16% in 2016, whereas macroalgae cover increased to ~30% in 2016. Both groups showed high spatiotemporal variability. Hard coral cover declined in total by 12% from 1978 to 2004 but increased again by 5% between 2005 and 2016 indicating some coral recovery after the 2005 mass bleaching event and hurricane impacts. In 2016, more than 80% of studied reefs were dominated by macroalgae, while only 15% were dominated by hard corals. This stands in contrast to 1978 when all reef sites surveyed were dominated by hard corals. This study is among the first within the Caribbean region that reports local recovery in coral cover in the Caribbean, while other Caribbean reefs have failed to recover. Most Mexican Caribbean coral reefs are now no longer dominated by hard corals. In order to prevent further reef degradation, viable and reliable conservation alternatives are required.

## Introduction

Monitoring change in coral reef ecosystems is essential in an era when humanity is having a widespread and long-term impact on nature. Current anthropogenic climate change and local stressors (such as overfishing and a mix of pollution and sedimentation from coastal development^1^) place coral reefs as the most endangered ecosystems on earth^2^. Rapid reversals in their health have been reported globally^3^, including reefs from the Caribbean region, where declines of the live hard coral cover of ~80% between 1975 and 2000 have been documented^4–6^. In the late 1970s, entire populations of reef-building coral species (i.e. *Acropora palmata* and *Acropora cervicornis*) collapsed as a result of the white-band disease^7^. Furthermore, the mass mortality of black sea urchins (*Diadema antillarum*), overfishing and eutrophication^8^ have resulted in a proliferation of more opportunistic, fast-growing organisms such as (macro)algae that outcompete reef-building corals^8–11^. As a result, many Caribbean benthic coral reef communities changed drastically from low coral cover to persistent states of high cover (macro)algae in the process of so-called phase shifts^11–15^. Efforts to mitigate or reverse phase shifts and reef degradation in the Caribbean include the development of new coral reef monitoring and managing strategies^16–18^.

**Figure 1:**
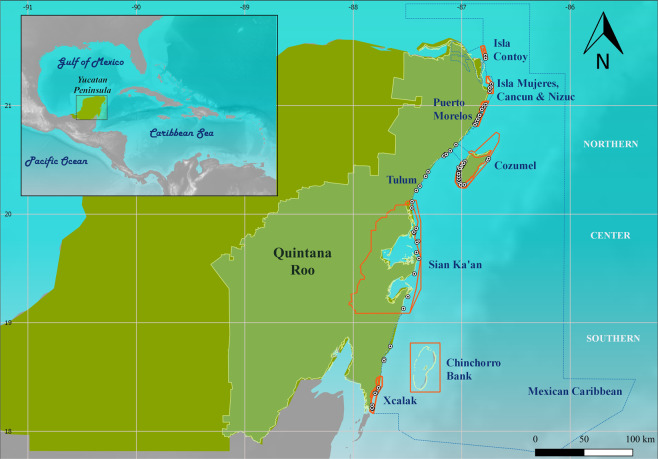
Map of the study area (made with QGIS Development Team, 2019. QGIS Geographic Information System. Open Source Geospatial Foundation. http://qgis.org). Polygons in orange represent the MPAs in the region (CONANP, Comisión Nacional de Áreas Naturales Protegidas (2019). Retrieved from https://datos.gob.mx/busca/organization/conanp). The Mexican Caribbean Biosphere Reserve was decreed in 2016, after this project period of analysis, therefore is represented with a blue dotted line. ◉ represent the monitoring sites.

Monitoring efforts of Caribbean reefs began in the late 1970s at various reef locations for short durations^19^. It was until 1980 when coral reef monitoring programs first began for some countries due to the evident reef degradation and increasing threats^19^. In the Mesoamerican Reef System (MAR), the monitoring officially began in 2005 with the Healthy Reefs for Healthy People Initiative^20^. The MAR is recognized by the World Wildlife Fund (WWF) as one of 200 global priority ecoregions whose biodiversity protection is essential^21^. This ecoregion within the Caribbean spans 1600 km along the coasts of Mexico, Guatemala, Belize, and Honduras and has experienced rapid changes within the last decades^22,23^. In the Mexican part, in particular, in Cozumel and the northern part of Quintana Roo (Fig. 1), the 2005 bleaching event and subsequent hurricane impacts affected more than 50% of coral colonies^19,24^. In 2007, hurricane Dean (category 5) hit the Southern Quintana Roo reefs affecting Mahahual and Chinchorro Bank^19^. In the following years, i.e., 2009–2011 and 2014–2017, Mexican Caribbean (MC) coral reefs were less affected by increasing sea surface temperatures (SST)^25^ and hurricane impacts^26^. Nonetheless, the rapid increases in macroalgae and the growing local threats diminish the capacity of the coral reefs to recover^11^.

Reefs in the MC are threatened since the establishment of Cancun as an international tourist destination in the 1970s^27^. Tourism industry rapidly expanded through the region and has impacted reefs and other ecosystems through the constructions of piers to receive massive tourist cruise ships^28^, the clearing of vegetation to construct roads, houses, restaurants and hotels^29,30^, and recreational activities. Recreational activities provide the leading services for tourism industry^31^. However, they also pose potential threats to reef communities, e.g. breakage of corals by divers and snorkelers, trampling, taking fishes for aquaria, oil contamination due to shipping, among others^32^. Indeed, there are additional threats of the MC coral communities such as invading species (e.g. lionfish [*Pterois volitans*])^33^ and the recent large floating mats of Atlantic *Sargassum* species reaching Western Caribbean coasts^34^. The disintegration of these *Sargassum* mats not only releases nutrients and consumes oxygen, but also decreases light availability at the seafloor, thereby affecting ecosystem functions such as benthic photosynthesis^35^. All these threats impacted MC reefs, but their extent is still unknown.

Taken together, the current degradation of MC reefs makes it necessary to set a detailed knowledge baseline of its ecological history in a regional context, e.g. changes in coral and macroalgae cover. Even though monitoring and research efforts have been regularly conducted in the MC since the early 1980s, this only permitted to roughly understand the history of change for this region^20^. Despite existing efforts, a quantitative long-term spatiotemporal analysis of the condition of MC reefs is still lacking. Hard coral and macroalgae development trends are essential to understand the current status of these reefs and to identify patterns of occurred changes. In the last decade, meta-analysis has been a widely used tool applied in coral reef studies, since it allows to systematically combine a wide range of information including monitoring and experimental field exploration to provide an integrative view across time^36^. Here, a meta-analysis of data from monitoring programs, peer-reviewed scientific publications, and grey literature was performed to describe the large-scale and long-term changes in MC coral reefs. This study aims to answer the following questions: 1) What is the extent of hard coral and macroalgae (calcareous and fleshy) benthic cover change in the MC over the last 38 years? 2) Are there temporal and spatial patterns of change?

## Results

### Hard coral and macroalgae cover between 1978 and 2016

**Figure 2:**
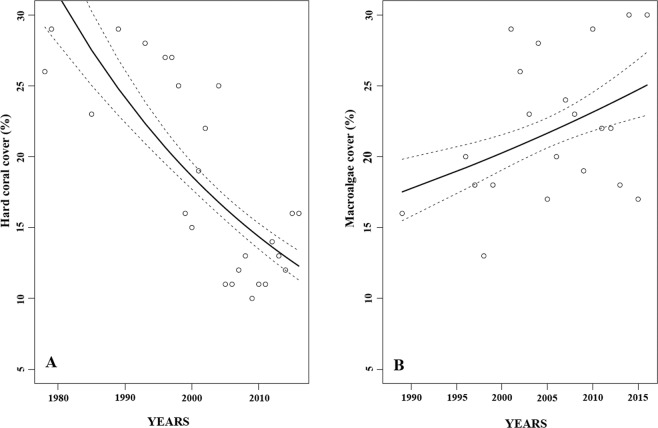
Annual means of benthic cover. **(A**) Hard coral from 1978 to 2016 and (**B**) macroalgae cover from 1989 to 2016. The solid line represents the regression line calculated from the estimates of a GLM with Gamma distributed error and log-link function, while the dotted lines represent the upper and lower 95% confidence interval.

**Figure 3:**
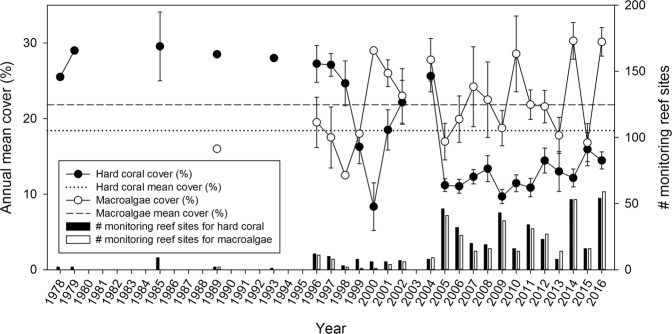
Annual means of hard coral and macroalgae cover (%) from 1978 to 2016. Circles represent hard coral cover (black), and macroalgae cover (white) as means ± S.E. (unless n < 3). Circles connected by the line represent subsequent years. The dotted and dashed lines represent the average hard coral cover and macroalgae, respectively. Bars represent the number of monitoring reef sites for hard coral (black) and macroalgae (white).

**Figure 4:**
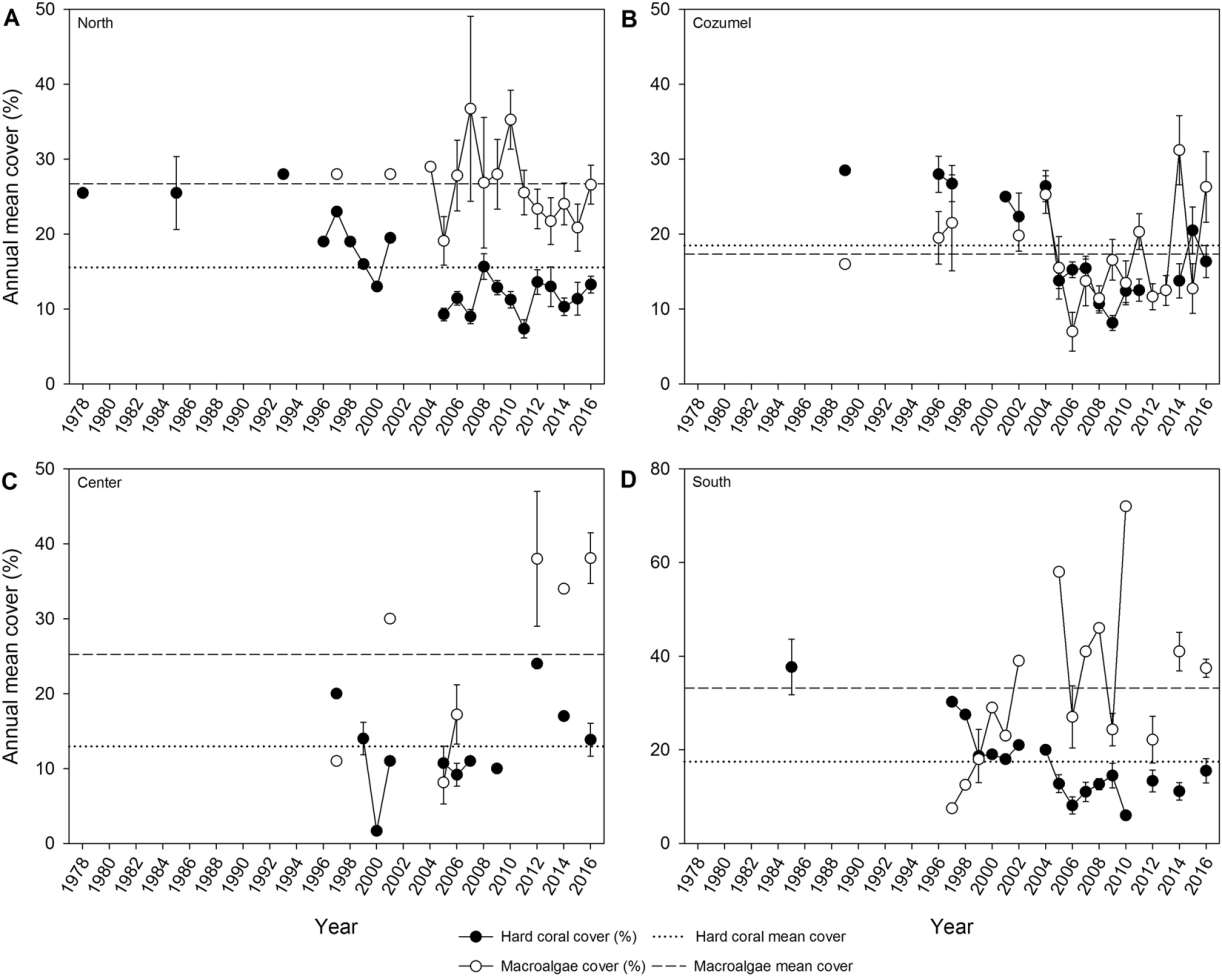
Regional annual means of hard coral and macroalgae cover (%) from 1978 to 2016. (**A**) The Northern region, (**B**) Cozumel, (**C**) Center, and (**D**) Southern region. Circles represent hard coral cover (black), and macroalgae cover (white) as means ± S.E. (unless n < 3). Circles connected by the line represent subsequent years. The dotted and dashed lines represent the average hard coral cover and macroalgae, respectively, in the studied time.

When considering the effect of time on the cover percentage, the region-wide hard coral cover declined by more than half during the last 38 years (from ~30% in the 1970s to ~12% in 2016; *p* < 0.0001; Figs. 2A and 3). In contrast, macroalgae cover increased for the study region from ~17% in the late 1980s to ~25% in 2016 (*p* < 0.05; Fig. 2B and 3). In 2005, hard coral cover and macroalgae cover were lower compared to the previous available data point, except for hard coral cover in the Center region (Fig. 4C). Whereas macroalgae increased and/or stabilized in subsequent years, hard coral cover only showed modest signs of recovery, especially in the Cozumel and Center regions, however maintaining low coverage during the following years (Fig. 4B and 4C).

The spatial patterns of hard coral and macroalgae cover present some differences between regions (Fig. 4). Around the 2000s, macroalgae became the dominant biotic component in the North, Center, and South subregions, with a mean cover close to 30% (Fig. 4). While the hard coral cover was consistently low (<15%) for the North and South subregions; data for the Center of the MC was more sparse and was not possible to depict clear temporal trends for any of the two variables. However, for this region, macroalgae cover has been consistently and significantly higher than hard coral cover since 2010. For the Cozumel subregion, temporal trends show a decline, for both macroalgae and hard coral cover in 2006, and subsequently a slight recovery for both variables. Cozumel is the only subregion in which the cover of macroalgae and hard coral have not yet diverged; nevertheless, macroalgae cover peaked in 2014 and 2016.

### Meta-analysis: temporal rates of change for the overall MC

**Figure 5:**
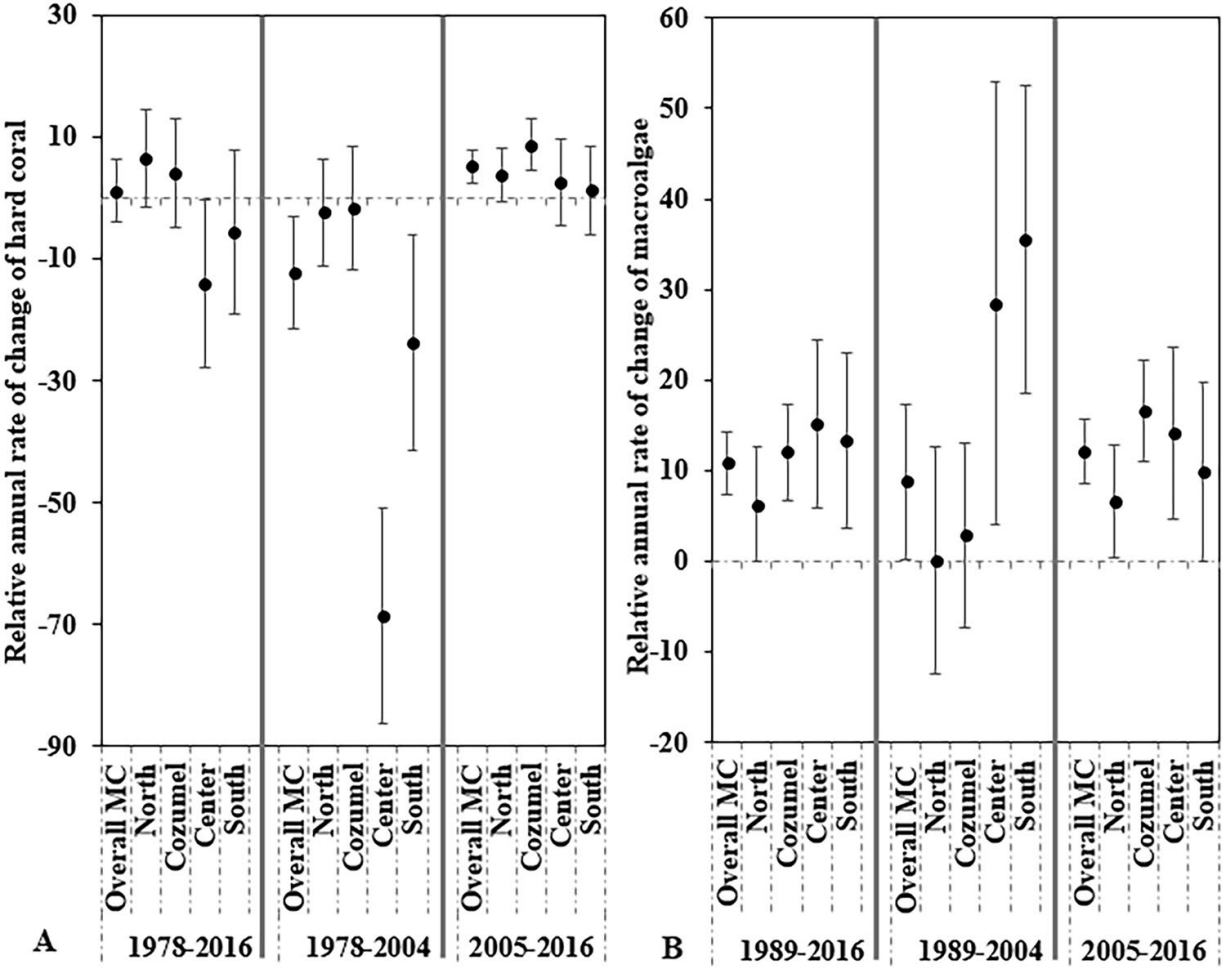
Mean effect sizes of the relative annual rate of change (ARC) for (**A**) hard coral cover and (**B**) macroalgae cover from the weighted random meta-analysis by regions and periods of analysis: 1978–2016, 1978–2004, and 2005–2016. The ARC mean effect sizes are presented with 95% confidence interval for separate analysis of cover change. The zero lines indicate no effect and the significance of ARC effects is determined when the 95% confidence interval does not overlap zero.

Temporal patterns of hard coral change varied along the two analyzed periods for the overall MC, presenting trajectories of decline and recovery (Fig. 5). The mean effect size for annual rate of change (ARC) in the hard coral cover decreased over the first period (1978–2004) and was significantly negative (ARC = ~−12%, *p* = 0.0094, n = 35; Fig. 5A), meaning that on average there was a net loss of hard coral cover in the overall MC. Conversely, the annual rate of change in the second period (2005–2016) was 5% (*p* < 0.001, n = 92) for the overall MC (Fig. 5A), meaning that there was a slight recovery in hard coral cover. However, when combined, the mean effect size for ARC remained stable throughout the entire study period (ARC = ~1%, *p* = 0.656, n = 125; Fig. 5A). The macroalgae mean effect size for ARC presented an increase in the whole period analyzed (1989–2016) by ~11% (*p* < 0.0001, n = 94; Fig. 5B). See Supplementary Table S1 and S2 for all spatiotemporal statistics.

### Spatiotemporal rates of change for each subregion

#### Northern region

In the Northern region, temporal differences in the mean effect size for ARC were found only for macroalgae, while the mean effect size of ARC for hard coral cover remained stable (Fig. 5A). In the whole period analyzed (1989–2016), the mean effect size for ARC for macroalgae cover was ~6%, (*p* = 0.0489, n = 29; Fig. 5B). Similarly, during the last period (2005–2016), the mean effect size for ARC for macroalgae cover was also ~6% (*p* = 0.0365, n = 28; Fig. 5B).

#### Cozumel sub-region

Temporal differences in the hard coral cover were found only for Cozumel over 11 years (2005–2016), where the mean effect size for ARC was ~9% (*p* < 0.0001, n = 35; Fig. 5A). In contrast, the mean effect size for ARC during the whole study period (1989–2016) for macroalgae was positive (~12%, *p* < 0.0001, n = 40; Fig. 5B). Similarly, for the period of 2005–2016 the mean effect size for ARC was also positive (16%, *p* < 0.0001, n = 34; Fig. 5B).

#### Center region

For the whole period analyzed (1978–2016) in the Center region, the mean effect size for ARC on hard coral cover was negative (~−14%, *p* = 0.045, n = 17; Fig. 5A). Likewise, the temporal analysis from 1978–2004 revealed a negative mean effect size for ARC on hard coral cover of ~−68% (*p* < 0.0001, n = 4; Fig. 5A).

In the period 1989–2016, the macroalgae mean effect size for ARC presented an overall increase of ~15% (*p* = 0.0014, n = 13; Fig. 5B). Temporal analysis also revealed significant differences. In the period 1989–2004, the macroalgae mean effect size for ARC presented a positive increase of ~28% (*p* = 0.02, n = 2; Fig. 5B), whereas in 2005–2016 it increased by ~14% (*p* = 0.0034, n = 12; Fig. 5B).

#### Southern region

In the Southern region, the mean effect size for ARC on hard coral cover for the first period analyzed (1978–2004) was negative (~−23%, *p* = 0.0082, n = 4; Fig. 5A). In contrast, the mean effect size for ARC on macroalgae cover increased over the whole period of study by ~13% (*p* = 0.007, n = 12; Fig. 5B). Additionally, the macroalgae mean effect size for ARC in 1989–2004 increased by ~35% (*p* < 0.0001, n = 2; Fig. 5B). Finally, from 2005 to 2016, the macroalgae mean effect size for ARC continued increasing by ~10% (*p* = 0.0506, n = 11; Fig. 5B).

## Discussion

Our meta-analysis of ecological changes in the Mexican Caribbean showed that, on a regional scale, hard coral cover experienced a steady rate of decline between 1978 and 2004, mainly driven by hard coral cover loss in the Center and South regions, and a slow relative increase in the second period (2005–2016), mainly driven by Cozumel (Fig. 5A). On the contrary, macroalgae cover consistently increased across time and for most of the subregions in the MC (Fig. 2B and 5B). However, different trajectories of change for both hard coral and macroalgae were found with high spatiotemporal variability (Fig. 5). It is important also to mention that regional trends in hard coral cover during the late 1970s and 1980s were of low resolution compared to the period of 2000s onwards because of the earlier lack in monitoring efforts (Fig. 3). Historically, the Northern and Cozumel regions were most exhaustively sampled over time, whereas in the Center and Southern regions, fewer monitoring surveys were performed. The Center and South regions experienced the greatest rates of hard coral cover decline and highly significant increases of macroalgae cover between 1978 and 2004. For the second period (2005–2016), the same regions did not show signs of hard coral recovery; and as in the rest of the regions, macroalgae cover continued on an increasing trend (Fig. 5B). A different trend was observed for Cozumel and the North regions. Between 1978 and 2004, only non-significant declines were observed for hard coral cover (Fig. 5A), and macroalgae cover remained relatively stable in these regions (Fig. 5B). Yet, it was not until the second period where the increase of macroalgae became more evident in the Cozumel and North regions.

Since macroalgae compete with corals for space^37^, macroalgae in the overall MC likely rapidly proliferated in response to declining hard coral cover. Phase shifts can occur in response to various factors, including the loss of crucial herbivores and eutrophication that enhances benthic algal biomass^38^. The results presented here reveal a phase shift from hard coral towards macroalgae domination in the overall MC (Fig. 3) with high and significant variability among regions and periods of analysis (Fig. 4). Certain conditions can modulate macroalgae increase, such as nutrient enrichment, especially in reefs located near highly populated areas^39^. Surprisingly, in the current study, the meta-analysis revealed a higher macroalgae increase in the more sparsely populated Center and Southern regions between 1989 and 2004 (Fig. 5B). Between 2001 and 2013, the Southern region has been subject to ~90% of forest cover loss due to human settlement, agriculture, and livestock farming^40^, which may have caused increased sediment run-off. High sediment run-off can affect corals through increasing suspended sediment and nutrient levels thus favoring macroalgae growth^41^.

Phase shifts from coral towards algae-dominated states have been widely reported in the Caribbean^42^. From 1978 to 2004, results reported here suggest that, on average, hard coral cover declined across the MC, and macroalgae increased (Fig. 5). These results correspond with what was found in other local-scale and regional studies in the Caribbean^4,5,43,44^. Different successive biotic and abiotic impacts in the wider Caribbean may explain these outcomes. First, the diseases of hard coral species (i.e., *A. palmata* and *A. cervicornis*)^7^ impacted the overall coral coverage and compromised the architectural complexity of these reefs^45^. Secondly, the hurricane impacts, especially in shallow reefs, changed the physical structure of the benthos, as well as the local species distribution, and habitat diversity^46^. However, macroalgae colonization in the MC is likely to be related to mortality events after the 1998 ENSO, resulting in mass bleaching and exacerbated by hurricane impacts, when opportunistic macroalgae began to colonize the free available space and continued increasing in MC reefs (Fig. 2). Our findings suggest that the phase shift from hard  coral towards macroalgae domination in MC started around the mid-2000s (Fig. 3). First, an overall increase in macroalgae and a decrease in hard coral cover, and later, a marked phase shift from hard coral to macroalgae domination, where hard coral cover halved, and macroalgae cover increased (Fig. 2). This regional-scale phase shift corresponds with the timing reported by previous studies that have assessed recent ecological changes at a local scale in the MC^47,48^.

Following the 2005 bleaching event and hurricane impacts, hard coral and macroalgae cover in the overall MC initially responded with similar declines in absolute cover, while their long-term responses displayed different trajectories defined by the fast (i.e., 11 years) recovery of macroalgae during the second period of study (2005–2016; Fig. 3). Despite this increase in macroalgae cover, in the second period, hard coral cover in the overall MC exhibited a modest recovery (i.e. 5%; Fig. 5A). This increase was driven mostly by the Cozumel region as this region was the only one where the mean effect size of ARC was significantly positive (Fig. 5A). However, the average hard coral mean effect size of ARC for the Northern, Center, and Southern regions were all positive thus supporting the mean effect size for ARC for the overall MC towards a positive trend (2005-2016; Fig. 5A). Generally, it is more common to find recovery rates after mass mortality events in areas far away from anthropogenic impacts such as the Seychelles islands^49^ and the archipelagos in the Central Pacific Ocean^50^. Here, the coral settlement, calcification, and reproduction has been successful in comparison with reefs located near highly populated areas. However, current investigations in the Chagos Archipelago report that coral growth still did not recover completely from warming events, thus keeping reefs in a low coral cover state^51^. Likewise, in remote islands in the Central Equatorial Pacific, corals and reef fish species biomass were severely reduced after the 2015/16 bleaching event^52^. In the Great Barrier Reef, the coral community was recovering at a slow growth rate compared with previous bleaching events and pre-disturbance status^53^.

MC reefs are directly exposed to chronic stressors. However, this study is one of the first within the Caribbean region that reports recovery. In the last decades, other Caribbean coral reefs have failed to recover after bleaching events^54,55^. This recovery does not necessarily suggest that the assemblage has recovered or maintained its previous diversity richness. Moreover, the recovery reported here, 5% between 2005 and 2016, is still less than half of what was lost in 2005 and is slow compared with recovery rates found in reefs of the Pacific after widespread mortality^49,56,57^. There are three potential explanations for the unexpected recovery in MC reefs: 1) The protection due to 13 Marine Protected Areas (MPAs) in the MC may have promoted the reported recovery^47,58^, 2) these management actions may have helped to increase resilience of MC reefs^59^, and 3) the dynamic of the water circulation and currents especially in the Northern region^60^ may help to flush and diminish the adverse effects of land-based pollution sources^61^.

The Northern region of the MC had experienced more rapid rates of coastal modification since the 1970s when Cancun was conceived as an international tourist destination^62^. Furthermore, this region has, potentially, the highest pressure of coastal development in the MC^63^. Surprisingly, this region had the lowest macroalgae increase (i.e., ~6%) for the entire study period (Fig. 5B). Three reasons may explain this result: first, this region had an older history of reef degradation; the reefs were already impacted by the time the first surveys began^64^. Second, the MPAs actions helped to conserve those systems during the last 20 years, and coupled with rehabilitation efforts, may have helped the reefs inside of the reserves to become more resistant^47^. One key example is Limones reefs in Puerto Morelos National Park which is the Cancun area of influence but also has the highest coral cover (mainly *A. palmata*) within the Northern region^65^. Third, the geology and local oceanographic conditions in the Northern region favor the formations of seasonal eddies^66^. Recirculating eddies may damage macroalgae production because of their velocities and turbulence^67^. Also, eddies favor coral larval retention and recruitment patterns^68^.

Coral reefs in Cozumel presented ~9% hard coral cover increase from 2005 to 2016. The high hard coral cover in this area suggests a higher resilience, even though extensive unsustainable tourism activities have taken place since the late 1970s. The creation of Cozumel as a National Park in 1980 was a significant effort that may have helped to build reef resilience and to cope with local anthropogenic impacts^69^. The best-developed reefs in this area are located in the leeward of Cozumel coast. According to Fenner^70^, the reef development in Cozumel in a sheltered area permitted a better reef growth. Regardless of the good shape of Cozumel reefs, macroalgae cover remains high as in the rest of MC reefs (Fig. 4B) and exhibits an increasing trend (Fig. 5B) similar to other reefs around the globe^71^. This may be attributed to increasing nutrient uptake on the island due to the human population growth in the last decades^72^. However, water quality studies are still lacking.

The Center area in the MC encompasses one of the largest protected areas in the region: The Biosphere Reserve of Sian Ka’an, with protection on the sea and land since 1986, experiencing little local anthropogenic impacts^73^. Surprisingly, coral reefs in this area experienced the most drastic decrease in hard coral cover and a substantial increase of macroalgae. Nevertheless, these results should be interpreted with caution due to the small sample size. Thus, for the first period analyzed (1978–2004), the sample size is reduced to 3 time-series studies. Observational studies noted the importance of the size of protected areas and stated that protected areas itself could improve coral reef health by protecting herbivorous fish, thus reducing macroalgae^74,75^. However, due to management measures in MC reefs, the herbivorous fish biomass has increased in the last decade^11^. Therefore, the most reliable explanation for the reported reef degradation in this area may be caused by the natural water circulation carrying nutrients and pollutants stressing the corals in different ways^60,76^. Gondwe *et al*.^77^ suggested through geochemical and phreatic analyses that Sian Ka’an has an entirely different groundwater system compared to, i.e., the Northern region. Indeed, systems surrounding Sian Ka’an experience a higher groundwater discharge of freshwater compared to the other coasts^76^. Moreover, this higher influx increases nutrient concentrations in the otherwise oligotrophic coastal wetlands of Sian Ka’an^78^. Consequently, nearby coral reefs may now be exposed to higher nutrient enrichment, sedimentation, and turbidity, resulting in a degraded state potentially favoring macroalgae overgrowth^39,79–82^.

Recent studies have described the Southern part as a focal point for mass tourism development without proper selective management strategies, possibly causing a more rapid increase in the macroalgae coverage^28,30^. As mentioned, herbivores are an essential biotic factor in regulating macroalgae^83^. However, this is not the case in these reefs. Evidence suggests that in the Southern region macroalgae increase is not related to a reduction in herbivores^48^. This may indicate that it did not increase sufficiently to control and restrict macroalgae growth, or that macroalgae are unpalatable. It has been suggested that different fish species have specific preferences for certain types of algae^84^, thus impacting the benthic community differently. Besides, critical grazers such as the sea urchin, *D. antillarum*, continues to be rare on most reefs since its region-wide die-off in the 1980s^85,86^, and although the populations of herbivorous fishes have increased, its biomass is likely far below historical baselines due to the impacts of decades of overfishing and habitat degradation^87^. Further anthropogenic local impacts, i.e. the pier construction (2000–2006) to receive massive cruise ships, fragmented the ecosystem facilitating coral degradation and overgrow of macroalgae. The lack of adequate management in the reefs in the Southern region pose higher local pressure to the coral reefs.

The current coral reef crisis is represented not only by a shift in species composition but also in the ecosystem functions and services^88^. Meta-analyses benefit coral reef research due to the synthesis of a large amount of data under specific objectives of the analysis. Primary information deriving from monitoring campaigns is highly valuable. However, if this information is not integrated and analysed into the bigger picture, likely, researchers will not be able to detect and predict the consequences of the occurred changes^89^. Moreover, identifying the significant factors impacting the coral reef health along the MC would be helpful to determine which areas require the most protection from anthropogenic activities. Even with the high number of MPAs and the creation of the MC MPA in 2016, monitoring active management and resources are still limited^20,90^. Meta-analyses enable long-term coral and algae cover assessment to set the baseline for a monitoring strategy based on science-based management that ensures coral reef biodiversity not only in the MC but also in the wider Caribbean.

In summary, coral disturbance regimes continue to intensify, while recovery capacities are now also dependent on human impacts^91^. The potential use of this study is to set the basis aimed to understand how the reefs have changed through time and what measures should be taken to respond and counter those changes. The use of individual studies integrated into a whole long-term longitudinal research can be used as a management tool. However, standardized future monitoring methods are highly recommended for the MC to follow up on the further development of these ecosystems.

## Material and Methods

### Study area

Reefs in the MC occur along the coast of the state of Quintana Roo (Fig. 1) and consist of various reef formations in terms of location, type and degree of development^20,92^. The MC can be divided into five main regions, i.e. Northern, Center, Southern, Cozumel, and Banco Chinchorro, each with its particular characteristics and features^20^. The Northern region spans from Isla Contoy to Tulum (Fig. 1). The fore reef zones in this area are generally flat and gently sloped^93^. It includes three MPAs, namely Isla Contoy, Puerto Morelos, the Costa Occidental de Isla Mujeres, Punta Cancun & Punta Nizuc. Cozumel region is an island also located in the North in front of the coastline of the Yucatan Peninsula (Fig. 1). The Center region is part of the Sian Ka’an Biosphere Reserve (Fig. 1), one of Mexico’s largest protected areas, which is a UNESCO world heritage site. The Southern region consists of ridges with a clear zonation of reef crest, front, slope, and terrace. This area also encompasses Arrecifes de Xcalak National Park (Fig. 1). The last region is Banco Chinchorro atoll and is also located in the Southern part (Fig. 1). However, due to a lack of monitoring information, it was omitted from the analysis. The MC region has an extensive network of MPAs that by 2016, nearly all the coral reefs have a protection status with the creation of the Mexican Caribbean Biosphere Reserve^20^.

### Data selection and extraction

The hard coral and macroalgae cover databases were collated by a wide variety of sources, including published literature, research protocols, grey literature, and monitoring programs (Supplementary Table S3 and S4). For this study, the category of macroalgae included both fleshy and calcareous macroalgae; many of the used sources did not report the type of macroalgae and combined them into one single group. Literature searches were conducted using standard search engines (e.g. ISI Web of Science and Google Scholar) using specific terms (e.g., coral reef AND hard*coral* OR coral* AND algae* OR macroalgae* AND benthic* AND Mexico* AND Mesoamerican*reef* AND Mexican*Caribbean*). All the information was curated, systemized and is now included in the Caribbean Reef Information System (CRIS), from the Biodiversity and Reef Conservation Lab, UNAM. The criteria for the potential inclusion of the study were as follows: (i) percentage cover of live hard coral and/or macroalgae; (ii) replicated measurements over time (not necessarily consecutive); (iii) if the authors use the same location of survey; (iv) the year of survey; (v) reports of the number and/or length of transects covered; and/or (vi) other variables e.g. water depth and reef zonation reported. If the studies reported monitoring and multi-temporal information, all sites defined in each study were used as a separate site. Care was taken not to double count coverage published in more than one study. If cover data were presented in graphical form, GetData^94^ was used to extract the percent cover. The raw monitoring information contributed a large number of sites to the dataset. The Northern region was sampled far more exhaustively than others (e.g. Center and Southern regions). Therefore, not all selected reef sites were surveyed in all years, although each reef site was visited at least two times. The monitoring database included 2,458 coral cover surveys on 125 reef sites between 1978 and 2016. Macroalgae cover was measured in 2391 surveys on 94 reef sites between 1989 and 2016. From all the included studies, 32% used the Atlantic and Gulf Rapid Reef Assessment (AGRRA) protocol to measure the benthic cover, 36% used the Synoptic Monitoring for the Mesoamerican Reef System (SAM) protocol, and 32% did not mention the use of a monitoring protocol but the use of other sampling methodologies (mainly Linear Intercept Transect). Both protocols focus on specific monitoring sites; one of the main differences is the methodology to monitor benthic organisms. SAM protocol uses point intercept methodology with 30 m length transects, whereas AGRRA protocol uses line intercept methodology with 10 m length transect^95,96^. The majority of data analyzed in this study was obtained during the same season of the year (May to October) following recommendations of the monitoring protocols (e.g. AGRAA, SAM).

### Data analysis

A regression analysis was performed to test the effect of time on overall yearly means of hard coral and macroalgae cover. Meta-analysis was used to analyze the temporal change in macroalgae and hard coral cover and how that change differed in the Northern, Cozumel, Center, and Southern regions of the MC.

### Regression analysis

The mean cover for hard coral and macroalgae for each year was calculated, pulling all the regions together. Since the cover of both the hard coral and the macroalgae were not normally distributed, the data were tested using a Generalized Linear Model (GLM) with Gamma error distribution and log link function, which best fit these data.

### Meta-analysis

Meta-analysis is one method of research synthesis supported by statistical procedures to merge the findings of individual primary studies (Supplementary Table S5^97^)^98^. The fundamental statistical parameter is the ‘effect size’. It standardizes the outcomes of different studies^99^, such that initially different measures can be combined and compared. There are different effect size methods to choose, depending on the availability of the data from primary studies^99,100^. Meta-analysis was used to detect overall changes at different spatial and temporal scales and sub-grouped by the three regions. Percentages of hard coral and macroalgae coverage for MC reefs were extracted from all available data that met the inclusion criteria. The random-effects meta-analysis was conducted in R using the “metaphor” package^98^. A random-effect model was used to represent the probability that any particular effect size is the best-approximating model to detect changes in coral reefs ecosystems. The relative annual rate of change (ARC) was the effect size used to measure the change in percentage cover for both hard coral and macroalgae percentage over time. Because of the principle of compounding, the annual rate of change is calculated over a period. In this study, the ARC is implemented by comparing the percentage cover in the same reef site at two different times to finally obtain an average mean for the period of interest, and it is computed as follows:

1 $${ARC}={(}{LogEnd}\,-\,{LogStart}{)}{/}{a}$$

where *Start* and *End* are the percentages hard coral cover or macroalgae at the start and end of the time series, respectively, *a* is the time in years elapsed between both measures^87,101^. Traditional meta-analysis weights within- and between-study sampling errors. However, the survey area has been found to produce more biologically realistic weightings for coral reef benthic data^5^.

For this reason, the weighting method for individual effect sizes was estimated by using the spatial area covered in each survey^101,102^. The mean effect size (MES) input for the meta-analyses are *yi*, which corresponds to the individual effect size per reef site and *vi*, which corresponds to the weighting method, defined as follows:

2 $${MES}{\approx }{rma}({yi}{,}{vi})$$

where *rma* is the function to fit the general linear models via mixed-effects in meta-analyses^98^.

All data points were pooled per year and averaged regardless of the method used in the surveys. The temporal heterogeneity was examined in two periods, before and after the 2005 mass bleaching event and hurricane impacts. The 2005 coral bleaching event was chosen as a cut-off point for data analyses because it was the warmest year in the Northern Hemisphere on average since reliable records in 1880^103^ and also because insufficient data were available for previous bleaching events in the MC (small sample size between 1978 and 2004). Reef monitoring efforts in MC increased considerably after this bleaching event. Because the individual effect sizes were log-transformed, they were back-transformed to percentages of coral/algae cover for interpretation purposes. Finally, a subset was made by grouping the surveyed sites by areas, i.e. (1) Northern, (2) Cozumel, (3) Center and (4) Southern (Fig. 1).

Previous research has established that independent effect sizes are a significant statistical premise of meta-analysis. Monitoring data are non-independent because various measures over time are conducted of the same experimental object. This non-independence is addressed in the meta-analysis by “treating each period as an individual study and the original studies as groups^104^.” Thus, each effect size constitutes a separate unit of information^100,105^. The data was ranked by the magnitude of effect size (independent of the direction) to assess the potential bias in this analysis. The largest effect size magnitude of each reef site was removed stepwise to define the number of studies that need to be removed to change the significance of the results^5^. If the exclusion of the largest effect size altered the significance of the results, that site was omitted from the analysis.

### Sensitivity analyses

Several analyses were performed to determine the meta-analysis’ sensitivity. The funnel plot is the most commonly used method to visually inspect the data^106^. This method assumes that the results from smaller studies will mainly spread around the bottom because of more substantial random error, and the more robust studies will spread towards the top^107^. In ecological studies, results are inclined to be published if they show significant effects^108^. In this regard, the database used includes a large sample of monitoring data and grey literature, so the sample of studies is not only drawn from those studies that were already published.

All data analyses were implemented and analyzed in the R Core Team (2018) software. The map of sites (Fig. 1) was produced with the software QGIS (Development Team, 2019). The remaining graphical representations were produced using R Statistics (R Development Core Team) and Sigmaplot 12.0 (Systat software) for Windows.

## Supplementary information

Supplementary information.

## Data Availability

Raw data of the current study are available from the corresponding authors on reasonable request.
