# Supplementary material for: A meta-analysis to assess long-term spatiotemporal changes of benthic coral and macroalgae cover in the Mexican Caribbean
Source: Sci Rep. 2020 Jun 1;10:8897. doi: 10.1038/s41598-020-65801-8 (PMC7264131; doi:10.1038/s41598-020-65801-8)
Supplement: Supplementary file 1 — Supplementary information. [file 41598_2020_65801_MOESM1_ESM.pdf]

Supplementary Information

A meta-analysis to assess long-term spatiotemporal changes of benthic coral and macroalgae cover in the Mexican Caribbean

Ameris I. Contreras-Silva[1*], Arjen Tilstra[1], Valentina Migani[2], Andra Thiel[2], Esmeralda Pérez-Cervantes[3], Nuria Estrada-Saldivar[3], Xochitl Elias[1], Claudius Mott[4], Lorenzo Alvarez-Filip[3*†], Christian Wild[1*†]

[1]Marine Ecology Department, Faculty of Biology and Chemistry, University of Bremen, Leobener Straße UFT, 28359, Bremen, Germany
[2]Population and Evolutionary Ecology Group, Institute of Ecology, Faculty of Biology and Chemistry, University of Bremen, Leobener Straße 5, 28359, Bremen, Germany
[3]Biodiversity and Reef Conservation Laboratory, Unidad Académica de Sistemas Arrecifales, Instituto de Ciencias del Mar y Limnología, Universidad Nacional Autónoma de México, Puerto Morelos, Quintana Roo, Mexico
[4]Remote Sensing Solutions GmbH, Dingolfinger Str. 9, 81673 München, Germany
*aics@unibremen.de, lorenzo@cmarl.unam.mx & christian.wild@uni-bremen.de
[†]These authors share senior authorship

Supplementary Table S1. Estimates resulting from the hard-coral meta-analyses divided by periods of time, general result and by sub-regions. ES denotes the mean effect size (ARC), n denotes the sample size, SE denotes standard error and *p* the significance of the statistical analysis.

	1978-2016				1978-2004				2005-2016			
	ES	SE	n	*p*	ES	SE	n	*p*	ES	SE	n	*p*
Overall MC	1.18	2.64	125	0.656	-12.16	4.68	35	0.0094	5.19	1.36	92	0.0001
North	6.47	4.12	50	0.1161	-2.40	4.51	16	0.5952	3.78	2.31	32	0.1022
Cozumel	4.05	4.55	41	0.3736	-1.67	5.45	11	0.76	8.75	2.18	35	<0.0001
Center	-14.15	7.06	17	0.0451	-68.55	9.04	4	<0.0001	2.59	3.56	13	0.4681
South	-5.58	6.86	18	0.4159	-23.82	9.01	4	0.0082	1.30	3.71	12	0.7272

Supplementary Table S2. Estimates resulting from the macroalgae meta-analyses divided by periods of time, general result and by sub-regions. ES denotes the mean effect size (ARC), n denotes the sample size, SE denotes standard error and *p* the significance of the statistical analysis.

	1989-2016				1989-2004				2005-2016			
	ES	SE	n	*p*	ES	SE	n	*p*	ES	SE	n	*p*
Overall MC	10.87	1.77	94	<0.0001	8.77	4.35	15	0.0437	12.06	1.84	85	<0.0001
North	6.28	3.19	29	0.0489	0	12.31	2	1	6.60	3.16	28	0.0365
Cozumel	12.05	2.72	40	<0.0001	2.87	3.73	11	0.44	16.55	2.87	34	<0.0001
Center	15.20	4.76	13	0.0014	28.51	12.44	2	0.02	14.10	4.82	12	0.0034
South	13.36	4.96	12	0.007	35.60	8.69	2	<0.0001	9.85	5.04	11	0.0506

Supplementary Table S3. Reef sites used for hard coral cover analyses. MPA (Marine Protected Area), Pub (origin of the information, 0 = monitoring or grey literature data; 1 = published data). NA = not available.

Num	*Site_code*	*Latitude*	*Longitude*	*Region*	*Municipality*	*Depth*	*MPA*	*Pub*
1	Akumal Garcia	20.398	-87.3052	Northern	Tulum	10	0	0
2	Akumal Garza	20.406	-87.30056	Northern	Tulum	10	0	0
3	Akumal Harvell	20.398	-87.3061	Northern	Tulum	NA	0	0
4	Akumal Rodriguez	20.383	-87.3151	Northern	Tulum	7	0	1
5	Boca Paila Garza	19.975	-87.4284	Center	F.Carrillo.Puerto	10	1	0
6	Bonanza	20.965	-86.81408	Northern	Puerto.Morelos	2	1	0
7	Cancun Barranco	21.181	-86.75833	Northern	Isla.Mujeres	NA	1	1
8	Cardona Mera Somero	20.408	-87.01955	Cozumel	Cozumel	5	1	0
9	Cardona ReefKeeper	20.414	-87.02031	Cozumel	Cozumel	NA	1	0
10	Chankanaab	20.44	-87.00295	Cozumel	Cozumel	12	1	0
11	Chankanaab Bolones	20.44	-87.00295	Cozumel	Cozumel	NA	1	0
12	Chankanaab Bolones Mera Profundo	20.44	-87.00512	Cozumel	Cozumel	18	1	0
13	Chankanaab Mera Somero	20.439	-86.99961	Cozumel	Cozumel	12	1	0
14	Chitales Jordan	21.141	-86.7437	Northern	Cancun	NA	1	1
15	Colombia	20.324	-87.02719	Cozumel	Cozumel	12	1	0
16	Colombia Mera Profundo	20.31	-87.02565	Cozumel	Cozumel	18.5	1	0
17	Colombia Mera Somero	20.32	-87.02437	Cozumel	Cozumel	6.5	1	0
18	Colombia ReefKeeper	20.326	-87.01681	Cozumel	Cozumel	NA	1	0
19	Cozumel Barranco	20.426	-87.0159	Cozumel	Cozumel	NA	1	1
20	Cozumel Garcia	20.329	-87.0269	Cozumel	Cozumel	10	1	0
21	Cuevones	21.162	-86.74199	Northern	Isla.Mujeres	7	1	0
22	Dalila	20.349	-87.02906	Cozumel	Cozumel	12	1	0
23	DzulHa Mera Somero	20.459	-86.98709	Cozumel	Cozumel	3	1	0
24	Hanan	20.505	-86.757	Cozumel	Cozumel	5	1	0

25	Hanan II	20.499	-86.761	Cozumel	Cozumel	8	1	0
26	Islote	20.441	-87.00233	Cozumel	Cozumel	15	1	0
27	Ixlache	21.435	-86.78	Northern	Isla.Mujeres	2	1	0
28	Jardines	20.833	-86.87844	Northern	Puerto.Morelos	2	1	0
29	La Bocana	20.875	-86.85172	Northern	Puerto.Morelos	4	1	0
30	La Pared	20.824	-86.87753	Northern	Puerto.Morelos	4	1	0
31	Las Redes_13	20.389	-87.31028	Northern	Tulum	13	0	1
32	Limones	20.988	-86.79719	Northern	Puerto.Morelos	3	1	0
33	Mah01	18.663	-87.71636	Southern	Othon.P.Blanco	11	0	0
34	Mahahual Arias	18.712	-87.70329	Southern	Othon.P.Blanco	15	0	1
35	Mahahual Garza	18.712	-87.70333	Southern	Othon.P.Blanco	10	0	0
36	Mahahual Harvell	18.723	-87.6971	Southern	Othon.P.Blanco	NA	0	0
37	Mahahual Rodríguez	18.805	-87.6583	Southern	Othon.P.Blanco	10	0	1
38	Media Luna_13	20.402	-87.30272	Northern	Tulum	13	0	1
39	MX1005	19.75	-87.40317	Center	F.Carrillo.Puerto	17	1	0
40	MX1006	19.829	-87.4399	Center	F.Carrillo.Puerto	18	1	0
41	MX1008	20.057	-87.46059	Center	F.Carrillo.Puerto	17	1	0
42	MX1010	20.348	-87.33246	Northern	Tulum	16	0	0
43	MX1017	21.171	-86.72976	Northern	Isla.Mujeres	6	1	0
44	MX1020	18.65	-87.71769	Southern	Othon.P.Blanco	11	0	0
45	MX1026	19.13	-87.53735	Center	F.Carrillo.Puerto	8	1	0
46	MX1028	19.239	-87.49639	Center	F.Carrillo.Puerto	14	1	0
47	MX1034	19.591	-87.39506	Center	F.Carrillo.Puerto	10	1	0
48	MX1035	19.74	-87.4135	Center	F.Carrillo.Puerto	6	1	0
49	MX1037	19.869	-87.4194	Center	F.Carrillo.Puerto	12	1	0
50	MX1042	20.115	-87.45794	Center	Tulum	9	1	0
51	MX1043	20.259	-87.38535	Northern	Tulum	8	0	0
52	MX1047	20.39	-87.31046	Northern	Tulum	10	0	0

53	MX1048	20.358	-87.02822	Cozumel	Cozumel	12	1	0
54	MX1050	20.536	-87.16451	Northern	Solidaridad	11	0	0
55	MX1053	20.486	-86.97072	Cozumel	Cozumel	5	1	0
56	MX1055	20.584	-87.10606	Northern	Solidaridad	7	0	0
57	MX1057	20.641	-87.05353	Northern	Solidaridad	2	0	0
58	MX1059	18.209	-87.82293	Southern	Othon.P.Blanco	7	1	0
59	MX1062	21.01	-86.77834	Northern	Isla.Mujeres	12	1	0
60	MX1065	18.353	-87.7907	Southern	Othon.P.Blanco	12	1	0
61	MX1066	21.46	-86.78122	Northern	Isla.Mujeres	7	1	0
62	MX1109	19.647	-87.41732	Center	F.Carrillo.Puerto	2	1	0
63	MX1116	20.551	-87.14924	Northern	Solidaridad	2	0	0
64	MX1117	20.218	-87.41906	Northern	Tulum	3	0	0
65	MX1131	20.916	-86.8288	Northern	Puerto.Morelos	4	1	0
66	MX1132a	20.987	-86.79642	Northern	Puerto.Morelos	2	1	0
67	MX1132b	20.987	-86.79642	Northern	Puerto.Morelos	2	1	0
68	MX1133	21.133	-86.74054	Northern	Isla.Mujeres	4	1	0
69	MX1134	21.199	-86.72548	Northern	Isla.Mujeres	6	1	0
70	MX1136	18.35	-87.79838	Southern	Othon.P.Blanco	1	1	0
71	MX2007	19.835	-87.44176	Center	F.Carrillo.Puerto	13	1	0
72	MX2033	19.45	-87.43655	Center	F.Carrillo.Puerto	11	1	0
73	MX2067	18.4	-87.76702	Southern	Othon.P.Blanco	10	1	0
74	MX3009	20.272	-86.99994	Cozumel	Cozumel	7	1	0
75	MX3021	18.783	-87.65809	Southern	Othon.P.Blanco	12	0	0
76	MX3054	20.511	-86.7524	Cozumel	Cozumel	12	1	0
77	MXXCK01	18.214	-87.82744	Southern	Othon.P.Blanco	9	1	0
78	MXXCK02	18.24	-87.82623	Southern	Othon.P.Blanco	7	1	0
79	Palancar Herradura	20.331	-87.02742	Northern	Cozumel	NA	1	0
80	Palancar Jardines Mera Profundo	20.334	-87.02722	Cozumel	Cozumel	22	1	0

81	Palancar Jardines Mera Somero	20.332	-87.0262	Cozumel	Cozumel	6	1	0
82	Palmas Mera Profundo	20.455	-86.99373	Cozumel	Cozumel	25	1	0
83	Paraiso	20.469	-86.98303	Cozumel	Cozumel	10	1	0
84	Paraiso Mera Somero	20.469	-86.98147	Cozumel	Cozumel	4	1	0
85	Paraiso Norte ReefKeeper	20.475	-86.97957	Cozumel	Cozumel	NA	1	0
86	Paraiso Sur ReefKeeper	20.472	-86.98232	Cozumel	Cozumel	NA	1	0
87	Paso del Cedral	20.374	-87.02894	Cozumel	Cozumel	13	1	0
88	Puerto Morelos	20.862	-86.8559	Northern	Puerto.Morelos	2	1	0
89	Puerto Morelos Harvell	20.847	-86.867	Northern	Puerto.Morelos	NA	1	0
90	Puerto Morelos Posterior Jordan	NA	NA	Northern	Puerto.Morelos	NA	1	0
95	PuertoMorelos Rodriguez	20.989	-86.7941	Northern	Puerto.Morelos	5	1	1
96	Punta Allen Rodríguez	19.784	87.4338	Center	Tulum	10	1	1
97	Punta Francesa Mera Profundo	20.357	-87.02963	Cozumel	Cozumel	17	1	0
98	Punta Francesa Mera Somero	20.362	-87.0272	Cozumel	Cozumel	7	1	0
99	Punta Maroma_10 Jordan	NA	NA	Northern	Solidaridad	10	0	0
101	Punta Maroma_Posterior Jordan	NA	NA	Northern	Solidaridad	5	0	0
104	Punta Nizuc_Posterior Jordan	NA	NA	Northern	Benito.Juarez	5	1	0
105	Punta Sur Mera Profundo	20.301	-87.02476	Cozumel	Cozumel	20	1	0
106	Punta Sur Mera Somero	20.298	-87.0194	Cozumel	Cozumel	4	1	0
107	Radio Pirata	20.854	-86.86501	Northern	Puerto.Morelos	1	1	0
108	San Clemente	20.408	-87.02197	Cozumel	Cozumel	8	1	0
109	San Francisco Mera Intermedio	20.397	-87.02603	Cozumel	Cozumel	12	1	0
110	Santa Rosa Bolones	20.377	-87.02933	Cozumel	Cozumel	17	1	0
111	Santa Rosa Mera Intermedio	20.378	-87.02849	Cozumel	Cozumel	11	1	0
112	Sta Rosa bajo	20.376	-87.02953	Cozumel	Cozumel	12	1	0
113	Tampalam Centro	19.146	-87.53611	Center	F.Carrillo.Puerto	20	1	0
114	Tampalam Norte	19.154	-87.53333	Center	F.Carrillo.Puerto	20	1	0
115	Tanchacte Norte	20.912	-86.83608	Northern	Puerto.Morelos	20	1	0

116	Tanchacte Sur	20.902	-86.84227	Northern	Puerto.Morelos	20	1	0
117	Tormentos	20.432	-87.01257	Cozumel	Cozumel	8	1	0
118	Tunich Mera Profundo	20.415	-87.0205	Cozumel	Cozumel	24	1	0
119	Uvero Harvell	18.952	-87.61	Southern	Othon.P.Blanco	NA	1	0
120	Xcalak Harvell	18.26	-87.8237	Southern	Othon.P.Blanco	NA	1	0
121	Xcalak Fore Steneck	18.32	-87.813	Southern	Othon.P.Blanco	13	1	1
122	Xcalak Garcia	18.264	-87.8233	Southern	Othon.P.Blanco	10	1	0
123	Xcalak Patch Steneck	18.265	-87.828	Southern	Othon.P.Blanco	2	1	1
124	Yalku Rodríguez	20.406	-87.2998	Northern	Tulum	10	0	1
125	Yucab	20.421	-87.01747	Cozumel	Cozumel	13	1	0

Supplementary Table S4. Reef sites used for macroalgae (calcareous and fleshy) analyses. MPA (Marine Protected Area), Pub (origin of the information, 0 = monitoring or grey literature data, 1 = published data). NA = not available.

Num	*Site_code*	*Latitude*	*Longitude*	*Region*	*Municipality*	*Depth*	*MPA*	*Pub*
1	Akumal Garcia	20.4	-87	Northern	Tulum	10	0	0
2	Akumal Garza	20.4	-87	Northern	Tulum	10	0	0
3	Boca Paila Garza	20	-87	Center	F.Carrillo.Puerto	10	1	0
4	Bonanza	21	-87	Northern	Puerto.Morelos	2	1	0
5	Cardona Mera Somero	20.4	-87	Cozumel	Cozumel	5	1	0
6	Cardona ReefKeeper	20.4	-87	Cozumel	Cozumel	NA	1	0
7	Chankanaab	20.4	-87	Cozumel	Cozumel	12	1	0
8	Chankanaab bolones	20.4	-87	Cozumel	Cozumel	NA	1	0
9	Chankanaab Bolones Mera Profundo	20.4	-87	Cozumel	Cozumel	18	1	0
10	Chankanaab Mera Somero	20.4	-87	Cozumel	Cozumel	12	1	0
11	Colombia	20.3	-87	Cozumel	Cozumel	12	1	0
12	Colombia Mera Profundo	20.3	-87	Cozumel	Cozumel	19	1	0
13	Colombia Mera Somero	20.3	-87	Cozumel	Cozumel	6.5	1	0
14	Colombia ReefKeeper	20.3	-87	Cozumel	Cozumel	NA	1	0
15	Cozumel Garcia	20.3	-87	Cozumel	Cozumel	10	1	0
16	Cuevones	21.2	-87	Northern	Isla.Mujeres	7	1	0
17	Dalila	20.3	-87	Cozumel	Cozumel	12	1	0
18	DzulHa Mera Somero	20.5	-87	Cozumel	Cozumel	3	1	0
19	Hanan	20.5	-87	Cozumel	Cozumel	5	1	0
20	Hanan II	20.5	-87	Cozumel	Cozumel	8	1	0
21	Islote	20.4	-87	Cozumel	Cozumel	15	1	0
22	Ixlache	21.4	-87	Northern	Isla.Mujeres	2	1	0
23	Jardines	20.8	-87	Northern	Puerto.Morelos	2	1	0
24	La Bocana	20.9	-87	Northern	Puerto.Morelos	4	1	0

25	La Pared	20.8	-87	Northern	Puerto.Morelos	4	1	0
26	Limones	21	-87	Northern	Puerto.Morelos	3	1	0
27	Mah01	18.7	-88	Southern	Othon.P.Blanco	11	0	0
28	Mahahual Arias	18.7	-88	Southern	Othon.P.Blanco	15	0	1
29	Mahahual Garza	18.7	-88	Southern	Othon.P.Blanco	10	0	0
30	MX1005	19.8	-87	Center	F.Carrillo.Puerto	17	1	0
31	MX1006	19.8	-87	Center	F.Carrillo.Puerto	18	1	0
32	MX1008	20.1	-87	Center	F.Carrillo.Puerto	17	1	0
33	MX1010	20.3	-87	Northern	Tulum	16	0	0
34	MX1017	21.2	-87	Northern	Isla.Mujeres	6	1	0
35	MX1020	18.6	-88	Southern	Othon.P.Blanco	11	0	0
36	MX1026	19.1	-88	Center	F.Carrillo.Puerto	8	1	0
37	MX1028	19.2	-87	Center	F.Carrillo.Puerto	14	1	0
38	MX1034	19.6	-87	Center	F.Carrillo.Puerto	10	1	0
39	MX1035	19.7	-87	Center	F.Carrillo.Puerto	6	1	0
40	MX1037	19.9	-87	Center	F.Carrillo.Puerto	12	1	0
41	MX1042	20.1	-87	Center	Tulum	9	1	0
42	MX1043	20.3	-87	Northern	Tulum	8	0	0
43	MX1047	20.4	-87	Northern	Tulum	10	0	0
44	MX1048	20.4	-87	Cozumel	Cozumel	12	1	0
45	MX1050	20.5	-87	Northern	Solidaridad	11	0	0
46	MX1053	20.5	-87	Cozumel	Cozumel	5	1	0
47	MX1055	20.6	-87	Northern	Solidaridad	7	0	0
48	MX1057	20.6	-87	Northern	Solidaridad	2	0	0
49	MX1059	18.2	-88	Southern	Othon.P.Blanco	7	1	0
50	MX1062	21	-87	Northern	Isla.Mujeres	12	1	0
51	MX1065	18.4	-88	Southern	Othon.P.Blanco	12	1	0
52	MX1066	21.5	-87	Northern	Isla.Mujeres	7	1	0

53	MX1109	19.6	-87	Center	F.Carrillo.Puerto	2	1	0
54	MX1116	20.6	-87	Northern	Solidaridad	2	0	0
55	MX1117	20.2	-87	Northern	Tulum	3	0	0
56	MX1131	20.9	-87	Northern	Puerto.Morelos	4	1	0
57	MX1132a	21	-87	Northern	Puerto.Morelos	2	1	0
58	MX1132b	21	-87	Northern	Puerto.Morelos	2	1	0
59	MX1133	21.1	-87	Northern	Isla.Mujeres	4	1	0
60	MX1134	21.2	-87	Northern	Isla.Mujeres	6	1	0
61	MX1136	18.3	-88	Southern	Othon.P.Blanco	1	1	0
62	MX2007	19.8	-87	Center	F.Carrillo.Puerto	13	1	0
63	MX2033	19.5	-87	Center	F.Carrillo.Puerto	11	1	0
64	MX2067	18.4	-88	Southern	Othon.P.Blanco	10	1	0
65	MX3009	20.3	-87	Cozumel	Cozumel	7	1	0
66	MX3021	18.8	-88	Southern	Othon.P.Blanco	12	0	0
67	MX3054	20.5	-87	Cozumel	Cozumel	12	1	0
68	MXXCK01	18.2	-88	Southern	Othon.P.Blanco	9	1	0
69	MXXCK02	18.2	-88	Southern	Othon.P.Blanco	7	1	0
70	Palancar Jardines Mera Profundo	20.3	-87	Cozumel	Cozumel	22	1	0
71	Palancar Jardines Mera Somero	20.3	-87	Cozumel	Cozumel	6	1	0
72	Palmas Mera Profundo	20.5	-87	Cozumel	Cozumel	25	1	0
73	Paraiso	20.5	-87	Cozumel	Cozumel	10	1	0
74	Paraiso Mera Somero	20.5	-87	Cozumel	Cozumel	4	1	0
75	Paraiso Norte ReefKeeper	20.5	-87	Cozumel	Cozumel	NA	1	0
76	Paraiso Sur ReefKeeper	20.5	-87	Cozumel	Cozumel	NA	1	0
77	Paso del Cedral	20.4	-87	Cozumel	Cozumel	13	1	0
78	Puerto Morelos	20.9	-87	Northern	Puerto.Morelos	2	1	0
79	Punta Francesa Mera Profundo	20.4	-87	Cozumel	Cozumel	17	1	0
80	Punta Francesa Mera Somero	20.4	-87	Cozumel	Cozumel	7	1	0

81	Punta Sur Mera Profundo	20.3	-87	Cozumel	Cozumel	20	1	0
82	Punta Sur Mera Somero	20.3	-87	Cozumel	Cozumel	4	1	0
83	Radio Pirata	20.9	-87	Northern	Puerto.Morelos	1	1	0
84	San Clemente	20.4	-87	Cozumel	Cozumel	8	1	0
85	San Francisco Mera Intermedio	20.4	-87	Cozumel	Cozumel	12	1	0
86	Santa Rosa Bolones	20.4	-87	Cozumel	Cozumel	17	1	0
87	Santa Rosa Mera Intermedio	20.4	-87	Cozumel	Cozumel	11	1	0
88	Sta Rosa bajo	20.4	-87	Cozumel	Cozumel	12	1	0
89	Tanchacte Norte	20.9	-87	Northern	Puerto.Morelos	20	1	0
90	Tanchacte Sur	20.9	-87	Northern	Puerto.Morelos	20	1	0
91	Tormentos	20.4	-87	Cozumel	Cozumel	8	1	0
92	Tunich Mera Profundo	20.4	-87	Cozumel	Cozumel	24	1	0
93	Xcalak Garcia	18.3	-88	Southern	Othon.P.Blanco	10	1	0
94	Yucab	20.4	-87	Cozumel	Cozumel	13	1	0

Supplementary Table S5. PRISMA 2009 Checklist

Section/topic	#	Checklist item	Reported on page #
**TITLE**			
Title	1	Identify the report as a systematic review, meta-analysis, or both.	1
**ABSTRACT**			
Structured summary	2	Provide a structured summary including, as applicable: background; objectives; data sources; study eligibility criteria, participants, and interventions; study appraisal and synthesis methods; results; limitations; conclusions and implications of key findings; systematic review registration number.	1
**INTRODUCTION**			
Rationale	3	Describe the rationale for the review in the context of what is already known.	2
Objectives	4	Provide an explicit statement of questions being addressed with reference to participants, interventions, comparisons, outcomes, and study design (PICOS).	2
**METHODS**			
Protocol and registration	5	Indicate if a review protocol exists, if and where it can be accessed (e.g., Web address), and, if available, provide registration information including registration number.	-
Eligibility criteria	6	Specify study characteristics (e.g., PICOS, length of follow-up) and report characteristics (e.g., years considered, language, publication status) used as criteria for eligibility, giving rationale.	7
Information sources	7	Describe all information sources (e.g., databases with dates of coverage, contact with study authors to identify additional studies) in the search and date last searched.	8
Search	8	Present full electronic search strategy for at least one database, including any limits used, such that it could be repeated.	7
Study selection	9	State the process for selecting studies (i.e., screening, eligibility, included in systematic review, and, if applicable, included in the meta-analysis).	7
Data collection process	10	Describe method of data extraction from reports (e.g., piloted forms, independently, in duplicate) and any processes for obtaining and confirming data from investigators.	-
Data items	11	List and define all variables for which data were sought (e.g., PICOS, funding sources) and any assumptions and simplifications made.	7

Risk of bias in individual studies	12	Describe methods used for assessing risk of bias of individual studies (including specification of whether this was done at the study or outcome level), and how this information is to be used in any data synthesis.	-
Summary measures	13	State the principal summary measures (e.g., risk ratio, difference in means).	7
Synthesis of results	14	Describe the methods of handling data and combining results of studies, if done, including measures of consistency (e.g., I^2) for each meta-analysis.	8
Risk of bias across studies	15	Specify any assessment of risk of bias that may affect the cumulative evidence (e.g., publication bias, selective reporting within studies).	9
Additional analyses	16	Describe methods of additional analyses (e.g., sensitivity or subgroup analyses, meta-regression), if done, indicating which were pre-specified.	9
**RESULTS**			
Study selection	17	Give numbers of studies screened, assessed for eligibility, and included in the review, with reasons for exclusions at each stage, ideally with a flow diagram.	3
Study characteristics	18	For each study, present characteristics for which data were extracted (e.g., study size, PICOS, follow-up period) and provide the citations.	-
Risk of bias within studies	19	Present data on risk of bias of each study and, if available, any outcome level assessment (see item 12).	-
Results of individual studies	20	For all outcomes considered (benefits or harms), present, for each study: (a) simple summary data for each intervention group (b) effect estimates and confidence intervals, ideally with a forest plot.	-
Synthesis of results	21	Present results of each meta-analysis done, including confidence intervals and measures of consistency.	3
Risk of bias across studies	22	Present results of any assessment of risk of bias across studies (see Item 15).	-
Additional analysis	23	Give results of additional analyses, if done (e.g., sensitivity or subgroup analyses, meta-regression [see Item 16]).	-
**DISCUSSION**			
Summary of evidence	24	Summarize the main findings including the strength of evidence for each main outcome; consider their relevance to key groups (e.g., healthcare providers, users, and policy makers).	4-7
Limitations	25	Discuss limitations at study and outcome level (e.g., risk of bias), and at review-level (e.g., incomplete retrieval of identified research, reporting bias).	6-7
Conclusions	26	Provide a general interpretation of the results in the context of other evidence, and implications for future research.	6-7
**FUNDING**			

Funding	27	Describe sources of funding for the systematic review and other support (e.g., supply of data); role of funders for the systematic review.	-
